# Deep Neck Space Infection in HIV-Infected Children: A Case Series

**DOI:** 10.7759/cureus.11081

**Published:** 2020-10-21

**Authors:** Kapila Hari, Sheetal Mungul, Shivesh Maharaj

**Affiliations:** 1 Internal Medicine, School of Medicine, University of the Witwatersrand, Johannesburg, ZAF; 2 Neurosciences, Charlotte Maxeke Johannesburg Academic Hospital, Johannesburg, ZAF; 3 Otorhinolaryngology, Charlotte Maxeke Johannesburg Academic Hospital, Johannesburg, ZAF

**Keywords:** hiv, paediatric deep neck space infections, antibiotics

## Abstract

Background

Deep neck space infections (DNSIs) in children may lead to airway compromise and damage to the great vessels in the neck. They occur more commonly in the HIV-infected population. To our knowledge, this is the first case series of DNSI in HIV-infected children

Objectives

The aim of this study was to describe the demography and document the sites of infection; organisms identified and resistance patterns in HIV-infected children with DNSI.

Methods

We retrospectively reviewed the clinical records of children (<16 years) diagnosed with deep neck infections at the teaching hospitals for the Department of Otolaryngology, Head and Neck Surgery, University of the Witwatersrand, between January 2010 and December 2018.

Results

We identified 17 patients with DNSI of which six children had concomitant HIV infection. The average age at presentation was six years (range: 0.35-13 years); there were four males and two females. The most common site involved was the submandibular space, which was affected in four patients. The detected organisms included: Coagulase-negative staphylococcus, Streptococcus viridans, Prevotella, Proteus mirabilis and Bacteroides fragilis. The organisms were universally resistant to penicillin and ampicillin resistance was documented in all but one patient.

Conclusion

Our findings on microbiology, resistance and tuberculosis culture are significant even in the face of a small series and have implications for the diagnosis and treatment of DNSI in HIV-infected children. Tuberculosis should routinely be considered in high burden settings. We recommend the empiric use of a β‐lactamase-resistant antibiotic until targeted therapy based on culture and sensitivity can be instituted.

## Introduction

The deep neck spaces are potential spaces constrained by cervical fascia. These spaces may be involved in head and neck infections. The term encompasses the submandibular, parotid, masticator, peritonsillar, parapharyngeal, retropharyngeal, pretracheal and prevertebral spaces. Infections originating in the ear, nose and/ or throat may spread to these spaces via contiguous or lymphatic spread resulting in abscess formation and subsequent life-threatening complications if left untreated [1]. The possible complications include upper airway obstruction, sepsis, mediastinitis jugular vein thrombosis and carotid artery rupture.

The management of deep neck space infections has progressed considerably in the last 100 years. Technological advances have improved diagnostic accuracy and targeted antimicrobial therapy has ensured the role of medical management as a viable treatment option. Human immunodeficiency virus (HIV)-related immunosuppression has been linked to rapidly progressive deep neck space infections (DNSIs) with resultant poor outcomes [1]. Low immunity in these patients may predispose to a longer recovery period and higher complication rates in comparison to HIV-uninfected individuals.

The incidence of DNSI has been shown to be higher in patients with underlying HIV infection [2]. A Taiwanese study quoted the incidence of DNSI in HIV-infected patients at around 57/10 000 people/year, a figure significantly higher than their control population (35.5/10,000). South Africa is the epicentre of the HIV pandemic. It is estimated that there are 340,000 (60,000-420,000) children aged 0-14years living with HIV in the country with 10,000 (8,100-20,000) new infections in 2019 [3]. New infections amongst South African children has declined, due to the successful implementation of the prevention of mother to child transmission programme. The scourge of HIV in South African children persists, however, with only 47% (36-58) of children receiving antiretroviral therapy (ART) [3].

Paediatric deep neck space infections are a distinct entity from adult deep neck space infections as lymph nodes are more predominant in different anatomical sites in children, resulting in different clinical presentations. Children are an especially vulnerable group as their ability to communicate symptoms is limited and this results in diagnostic challenges from a clinical perspective, which poses a risk for serious complications [4]. Bacterial organisms implicated in pediatric DNSI differ from those in adults [5]. The likely explanation for the difference is that adult deep neck space infections are usually due to dental pathology whilst pediatric deep neck space infections originate from various sources including tonsillitis, pharyngitis, hematogenous spread and suppurative cervical lymphadenitis [5].

We have previously documented the organisms and resistance patterns of paediatric DNSI in our setting [6]. In the face of recognized high levels of antibiotic resistance amongst HIV-infected children and resultant implications for the choice of empiric therapy we studied the demography and documented the organisms and resistance patterns in HIV-infected children with DNSI [7]. To our knowledge, this is the first case series of DNSI in HIV-infected children.

## Materials and methods

We retrospectively reviewed the clinical records of paediatric patients (<16years) diagnosed with deep neck infections at the Charlotte Maxeke Johannesburg Academic hospital for the Department of Otolaryngology, Head and Neck Surgery, University of the Witwatersrand, between January 2010 and December 2018. A list was compiled of all children admitted with DNSI involving the submandibular, parotid, masticator, peritonsillar, parapharyngeal, retropharyngeal, pretracheal and prevertebral spaces. Our policy is to perform surgical drainage in patients found to have a collection of pus on clinical or imaging examination; those with complications and those who did not respond to antibiotics within the first 48 hours.

A total of 17 children with deep neck space infection were tested for associated HIV infection as a predisposing factor. This is because most pediatric patients presenting to our institution already have a ‘Road to Health Card’, on which their maternal HIV exposure is documented. National policy for prevention of mother-to-child transmission necessitates all HIV-exposed babies to be tested and their status is documented on this ‘Road to Health Card’. Therefore, only in cases of uncertainty, where there is likely maternal HIV exposure with concern of mother to child transmission, is an HIV test repeated. 

The following information was recorded in each case: age and sex; CD4 and viral load (HIV positive children); anatomical spaces involved; imaging features; microbiology including sensitivities and resistance; complications and outcomes

Limitations

The main problem experienced was the lack of consistent record-keeping. 

Ethics

The study was part of the MMEd research report for Dr Sheetal Mungul. Ethics clearance was granted by the Human Research Ethics Committee (Medical) of the University of the Witwatersrand (clearance certificate number M180206). 

The guidelines of the Helsinki declaration were followed.

## Results

The demographic data for the six cases are recorded in Table 1. 

**Table 1:** Demographic data LDL, lower than a detectable level.

	Age (years)	Sex	Hb (g/dl)	CD4 (cells/mm^3^)	Viral Load	No. of days
Case 1	1.5	Female	11.1	2034	LDL	3
Case 2	7.0	Male	11.8	630	LDL	10
Case 3	13.0	Female	10.4(mod)	754	100	9
Case 4	0.35	Male	11.8	630	LDL	9
Case 5	4.5	Male	8.1(mod)	849	5705	6
Case 6	10.0	Male	13.5	1371	20	12

 

All six patients had both ultrasounds as well as CT scans of the neck during their hospital admission. The spaces involved included the submandibular space (n-4) and the parotid space (n-2).

The HIV-infected patients had an average CD4 count of 1044 cells/mm. In the patients with HIV, the viral load was noted to be lower than a detectable level (LDL) in four patients whilst the remaining two patients had viral loads of 5705 and 100. The HIV positive patients had an average hospital stay of 8.2 days. 

All patients required surgical drainage of their infection. The detected organisms included: Coagulase negative staphylococcus, Streptococcus viridans Prevotella, Proteus mirabilis and Bacteroides fragilis. Tuberculosis was identified in one patient. The sensitivity and resistance patterns of the identified organisms are documented below (Table 2). 

**Table 2:** Microbiology

	Neck site	Organism/s isolated	Organism/s sensitivities	Organism/s resistance	TB culture
Case 1	Sub-mandibular space	Coagulase negative staphylococcus	Penicillin	Nil	Negative
Case 2	Sub-mandibular space	Streptococcus viridans	Cefotaxime Ceftriaxone	Penicillin & Ampicillin	Negative
		Prevotella bivia	Amoxycillin & clavulanic acid	Penicillin & Ampicillin	
Case 3	Parotid space	Nil	N/A	N/A	Positive
Case 4		Streptococcus viridans	Cefotaxime Ceftriaxone	Penicillin	Negative
	Sub-mandibular space	Prevotella bivia	Amoxycillin & clavulanic acid	Penicillin	
Case 5	Sub-mandibular space	Nil	N/A	N/A	Negative
Case 6	Parotid space	Streptococcus constellatus	Penicillin, Ampicillin Cefotaxime	Nil	Negative
		Proteus mirabilis	Amoxycillin & clavulanic acid	Penicillin & Ampicillin	
		Bacteroides fragiis	Amoxycillin & clavulanic acid	Penicillin & Ampicillin	

## Discussion

In the face of an ongoing pandemic, unusual case presentations of HIV and its co-infections remain relevant in our clinical setting. This case series describes the clinical profile and bacteriology in six pediatric patients with DNSI. In our series, half of the patients were aged less than six years. Paediatric DNSI infections occur most commonly in children under the age of six years [8]. It is difficult to draw meaningful conclusions based on our small number of patients, but the preponderance of males is consistent with published data on pediatric DNSI [8]. The average hospital stay of 8.2 days is notably higher than the 5 days recorded in an equivalent HIV-uninfected population [6].

We have previously identified anaemia as a risk factor in the development of DNSI in children [6]. In the current study, only two patients had moderate anaemia, based on the World Health Organization (WHO) criteria [9]. In all but one of our patients, the HIV was well controlled, as evidenced by the viral load measurements. The effects of ART In reducing the morbidity and mortality associated with HIV infection is well established. It has however been shown that the risk of DNSI is higher amongst HIV-infected patients, even those with access to therapy [2]. 

The sub-mandibular space was involved In half of the patients in this series, suggesting underlying odontogenic infections as the precipitating factor in the development of the DNSI [10]. Periodontal disease is strongly associated with underlying HIV infection and this association is not linked to the stage of HIV infection [11,12]. The identification of Streptococcus Viridans and Prevotella Bivia in two of the patients with sub-mandibular space involvement is further affirmation of underlying odontogenic infection [13]. The microbiology of the organisms cultured is inconsistent with our previous data but the small number of cases makes further comment on specific organism culture superfluous [6].

Deep neck infections are a rare clinical presentation of tuberculosis infection [14]. The culture of the organism in one of our cases reflects the high incident rate of the disease in South Africa, which is one of 30 high burden countries in the world [15].

Antimicrobial resistance is a worldwide phenomenon, exacerbated in low socio-economic countries [16]. This is borne out in our limited case series. All organisms cultured were resistant to penicillin and most were resistant to ampicillin as well. This finding is important in that a recent review on DNSI advocated for the use of penicillin in combination with ampicillin/ clavulanic acid as empiric therapy [5].

## Conclusions

Our findings on microbiology, resistance and tuberculosis culture are significant even in the face of a small series and have implications for the diagnosis and treatment of DNSI in HIV-infected children. Tuberculosis testing should routinely be considered in high burden settings. We recommend the empiric use of a β‐lactamase-resistant antibiotic until targeted therapy based on culture and sensitivity can be instituted. Early detection and appropriate antibiotic management may lead to decreased in-hospital stays.
